# Steam Explosion Pretreatment for Improving Wheat Bran Extrusion Capacity

**DOI:** 10.3390/foods11182850

**Published:** 2022-09-15

**Authors:** Lan Wang, Tairan Pang, Feng Kong, Hongzhang Chen

**Affiliations:** 1 State Key Laboratory of Biochemical Engineering, Beijing Key Laboratory of Biomass Refining Engineering, Institute of Process Engineering, Chinese Academy of Sciences, No. 1 Bei-Er-Jie, Zhongguancun, Haidian District, Beijing 100190, China; 2 University of Chinese Academy of Sciences, No. 19(A) Yuquan Road, Shijingshan District, Beijing 100049, China

**Keywords:** steam explosion, wheat bran, dietary fiber, extrusion capacity

## Abstract

Extrusion improves the texture of wheat bran and enhances its product edibility, making it a promising processing method. However, the extrusion performance of wheat bran without any treatment is not satisfactory and limits the utilization of wheat bran in food processing. In this study, steam explosion pretreatment was used to treat wheat bran to investigate its promotion of wheat bran extrusion. The results showed that steam explosion could increase the extrusion ratio of wheat bran extrudate by 36%. Grinding the steam-exploded wheat bran extrudate yields wheat bran flour with smaller particle sizes and higher cell wall breakage. Fourier transform infrared spectroscopy and chemical composition results revealed that steam explosion degraded insoluble dietary fiber and disrupted the dense structure of the cell wall in wheat bran. The water-extracted arabinoxylan and soluble dietary fiber content of steam-exploded wheat bran were 13.95% and 7.47%, respectively, improved by 1567.42% and 241.75% compared to untreated samples. The total phenol and flavonoid contents, water solubility index, and cation exchange capacity of steam-exploded wheat bran extrudate were all superior to raw wheat bran extrudate. In summary, this study demonstrates that steam explosion improves the extrusion capacity of wheat bran and facilitates its utilization.

## 1. Introduction

Extrusion is a typical thermo-mechanical processing technology that processes raw materials at high temperatures with a short mechanical shear [1,2]. The extruded product has an expanded structure and ripening characteristics that attract consumers [3]. Extrusion can disrupt the physical structure of macromolecules (such as proteins and starches) into small molecules, thus changing the properties of the food ingredients and thereby improving the taste of the food and facilitating its digestion and absorption by the body [4]. However, extruded products generally have a high-calorie content, limiting their development [5].

Products with high dietary fiber content and low energy density are more acceptable to consumers [6]. Dietary fiber consists of cellulose, lignin, and hemicellulose (highly substituted insoluble arabinoxylan) [6,7]; it is beneficial to human health and improves intestinal health as well as prevents some diseases such as constipation and colon cancer [8]. Wheat bran, a by-product of wheat flour processing, is rich in dietary fiber and phenolic compounds and has potential use as a food product, which can positively affect the nutritional value of foods [9,10,11].

However, the high dietary fiber content in whole wheat makes extrusion challenging [6]. This phenomenon is mainly due to the insoluble dietary fiber in wheat bran, which affects starch extrusion [12]. Increasing the wheat bran content in the extrusion formulation can reduce the extrusion ratio, thus reducing the textural properties of the product [6,13]. In addition, wheat bran can cause the extruder to run erratically, leading to the machine being clogged or even exploding. Wheat bran contains 44–50% dietary fiber, most of which is insoluble [14], resulting in inferior bran extrusion properties. Numerous studies have pointed out that soluble dietary fibers provide better extrusion performance and more favorable textures of products than insoluble dietary fibers [6]. Thus, it is necessary to find ways to avoid this negative effect of insoluble dietary fiber in wheat bran.

Steam explosion is a green and efficient method that uses highly permeable saturated vapor to pretreat the material and release the pressure instantaneously to achieve a cell wall disruption effect [15]. Steam explosion is principally applied to the pretreatment of lignocellulosic materials (e.g., wheat straw, maize straw) and can destroy the structure of insoluble dietary fibers such as cellulose and hemicellulose [16]. This study, therefore, investigates the steam explosion treatment as a method to improve the expansion properties of wheat bran extrudates. The expansion rate and grindability of wheat bran before and after steam explosion were characterized. Subsequently, the effects of steam explosion and extrusion were evaluated on the chemical composition of wheat bran. Finally, the improvement of wheat bran in functional properties (water holding capacity, swelling, ion exchange properties) after the two treatments was also studied. This study provides a novel strategy for processing wheat-bran-based extruded foods and is expected to broaden the path for the utilization of wheat bran products towards efficient utilization.

## 2. Materials and Methods

### 2.1. Materials

Wheat bran was provided by Langfang Mountain Food Co., Ltd. (Hebei, China). The compositions of wheat bran were 37.6% starch and 48.6% total dietary fiber on a dry-weight basis. Heat-stable α-amylase, protease, and amyloglucosidase were purchased from Shanghai Macklin Biochemical Co., Ltd. (Shanghai, China). All other chemicals used in the experiments were of analytical grade.

### 2.2. Sample Preparation

Wheat bran was rehydrated with distilled water for about 30 min to adjust the initial moisture content to 30% (*w*/*w*). Steam explosion pretreatment was performed in a 10 L self-designed batch vessel that consisted of three units: a steam generator, a reaction chamber, and two reception chambers (Weihai Automatic Control Reactor Co., Ltd., Weihai, China), each controlled by a ball valve between the units. [1,17,18] Wheat bran was loaded into the reaction chamber, treated under 0.8 MPa of water vapor, and held for 5 min. Then, the discharge valve was opened to allow the reaction chamber to unload the pressure quickly, and wheat bran entered the receiving chamber. Raw and steam-exploded wheat bran samples were extruded in an extruder (SYSLG30-IV, Shan Dong Saibainuo Machinery Co., Ltd., Dezhou, China) at a moisture content of 33%, with a maximum temperature of 140 °C and a feeding speed of 15 Hz. Wheat bran extrudate was dried at 60 °C for 12 h, then packed in polyethylene bags and stored at 4 °C until analyzed.

### 2.3. Characterization

#### 2.3.1. Fourier Transform Infrared Spectroscopy

Fourier transform infrared spectroscopy (FTIR) was performed on the samples according to Zhao et al. [1] using an FTIR-8400S spectrometer (Shimadzu, Japan). Specifically, 1 mg of the dried sample was mixed with 100 mg of KBr and then pressed for 10 min at 8 tons to prepare the discs. Semi-quantitative analysis of the FTIR spectra, according to the method [2], and the band at 1519 cm^−1^ was used as a reference band to estimate the relative intensity of other bands [1].

#### 2.3.2. Chemical Composition Analysis of Wheat Bran

Dietary fiber in wheat bran samples was analyzed using the American Association of Cereal Chemists (AACC) method 32-07. Total arabinoxylans and water-extractable arabinoxylans content were determined using the method of Hashimoto et al. [3].

#### 2.3.3. Examination of Extrusion Ratio and Grinding Performance of Extruded Wheat Bran

The extrusion ratio of the extruded wheat bran was referenced in Zhu et al. [7] and Alam et al. [19], respectively. The die diameter used in the experiments was 3 mm. After grinding 100 g samples in an FW-400A grinder (Zhongxingweiye, Beijing, China) for 2 min, the particle size distribution was measured at room temperature using an NKT6100-B laser particle size meter (Naikete, Jinan, China). The particle size distributions and the particle sizes corresponding to the cumulative percentage of particle size distribution for samples reaching 10%, 50%, and 90% (D10, D50, and D90) were calculated by the software provided with the instrument [8]. The cell wall breakage ratio of samples was calculated using the method of Xiao et al. [9].

#### 2.3.4. Total Phenolic Content and Total Flavonoid Content of Extruded Wheat Bran

Total phenolic content was measured by the Folin–Ciocalteu method using gallic acid as the standard, as described by Wu et al. [4]. Absorbance was measured using a UV–vis spectrophotometer (UV-5800PC, Shanghai Metash Instruments Co., Ltd., China) at 760 nm against a reagent blank. The total phenolic content was expressed as milligrams of gallic acid equivalents per gram of dry sample weight (mg of GAE/g) through the calibration curve of gallic acid.

The total flavonoid content was determined using an aluminum chloride colorimetric method described by Jia et al. [6]. The absorbance was measured at 510 nm using a UV–vis spectrophotometer. The total flavonoid content was expressed as milligrams of catechin equivalents per gram of dry sample weight (mg of CAE/g) using the calibration curve of (±)-catechin.

#### 2.3.5. Expansion Capacity and Cation Exchange Capacity Detection of Extruded Wheat Bran

The expansion capacity of samples was determined using the method of Raghavendra et al., [10] water solubility index was measured as described by Jafari et al., [11] and cation exchange capacity was determined according to Ralet et al. [12]; a 0.25 g sample was taken in 10 mL of 0.1 mol/L HCl solution, placed at room temperature for 24 h, and then filtered to wash away the excess acid. The filtered residue was added to 100 mL 5% sodium chloride solution, and the solution was titrated with 0.01 mol/L sodium hydroxide solution. Instead of hydrochloric acid, distilled water was used to determine the sodium hydroxide consumed as a blank control. The cation exchange capacity was calculated by the following equation:

(1) $$CEC=\frac{\left(V_{1}-V_{2}\right)\times C}{m}$$

where *CEC* is the cation exchange capacity of the sample (mL/g), *V*_1_ is the volume of sodium hydroxide solution consumed by the sample (mL), *V*_2_ is the volume of sodium hydroxide solution consumed by the blank (mL), *m* is the mass of the sample (g), and *C* is the concentration of the sodium hydroxide solution (mol/L).

#### 2.3.6. Statistical Analysis

The results are reported as mean ± standard deviation of at least three replicated determination results. Experimental data were processed by one-way analysis of variance using IBM SPSS Statistics 20 (IBM, Armonk, NY, USA) with Duncan’s multiple range test (*p* < 0.05).

## 3. Results and Discussion

### 3.1. Functional Groups and Chemical Composition of Wheat Bran before and after Steam Explosion

**Figure 1:**
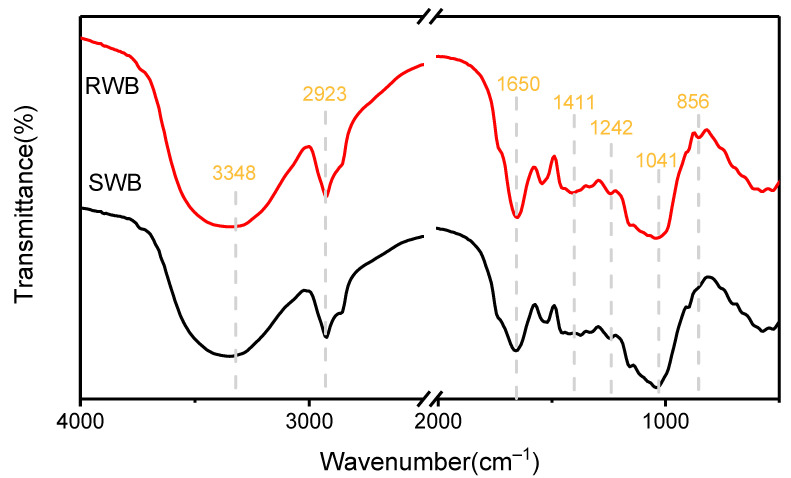
FTIR spectroscopy of raw wheat bran (RWB) and steam-exploded wheat bran (SWB).

FTIR spectra can effectively characterize the functional groups in raw materials and reflect the changes in the chemical structure of wheat bran before and after the steam explosion. Figure 1 illustrates the positions of the absorption peaks in the FTIR spectra of wheat bran before and after the steam explosion. The wavenumbers peaked at 3348 (OH), 2923 (CH), 1411 (CH_2_), 1242 (CO), and 1041 cm^−1^ (CO), indicating the presence of cellulose, hemicellulose, and lignin in wheat bran. The absorbance peak at 1242 cm^−1^ is associated with the C–O stretching vibration of the acetyl group in the lignin, and the peak at 1411 cm^−1^ is attributed to the bending vibration of the C–H group of the aromatic ring in the polysaccharides [20]. The band at 1041 cm^−1^ is owed to the C–O stretching modes of the hydroxyl and ether groups in the cellulose. The peak at 2905 cm^−1^ is a characteristic band for the C–H stretching vibration of methyl and methylene in the cellulose and hemicellulose components [21]. The final peak at 3345 cm^−1^ is owed to the presence of O–H stretching vibrations and the hydrogen bond of the hydroxyl groups. The reduction observed in the relative intensity of the 1041 cm^−1^ band after steam explosion suggests that steam explosion promotes the degradation of dextran and xylan to glucose and xylose, respectively [1]. The relative intensity of the peak at 1242 cm^−1^ decreased, suggesting that steam explosion reduced the relative content of the guaiacyl lignin units [1]. The spectra presented peaks at 1650 cm^−1^, corresponding to amide I in proteins. The result showed that steam explosion reduced the intensities of the amide I bands and might degrade protein [13]. The relative intensity of the 2923 cm^−1^ band, attributed to the C-H vibration of methyl and methylene [14], decreased from 1.2385 in raw wheat bran to 1.0782 in steam-exploded wheat bran (Table 1), indicating a reduction in the methyl and methylene components of steam-exploded wheat bran, further reflecting the reduced dietary fiber content [15,16]. The peak in this spectrum at 3348 cm^−1^ was associated with hydrogen and hydroxyl-bound O-H [22]. The decrease in the relative peak intensity of the steam-exploded wheat bran indicates a reduction in O-H bonds. The chemical structure of wheat bran was significantly altered after the steam explosion. Steam explosion pretreatment reduced the crystallinity of the cellulose in wheat bran and degraded the dietary fiber, thus promoting the expansion ratio and grindability of the extruded wheat bran [21].

**Figure 2:**
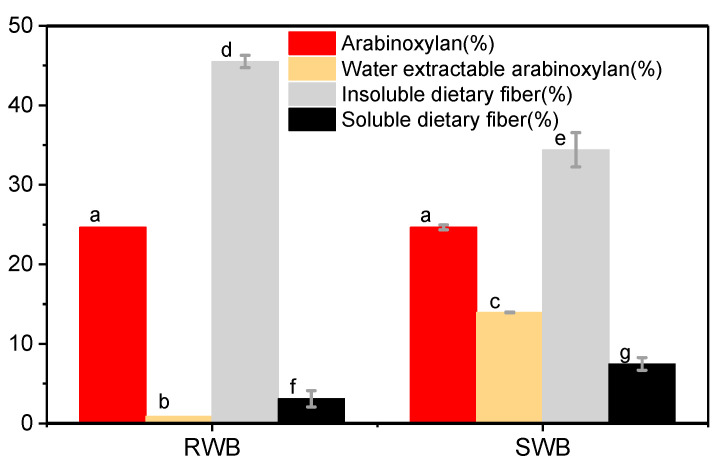
Chemical composition of raw wheat bran (RWB) and steam-exploded wheat bran (SWB); means that are significantly different (*p* < 0.05) are indicated by different letters.

The steam explosion and extrusion process can produce a series of chemical reactions that result in variations in the chemical and nutritional composition of the sample. The arabinoxylan, water-extractable arabinoxylan, insoluble dietary fiber, and soluble dietary fiber contents of samples are shown in Figure 2. It can be seen that the insoluble dietary fiber content of wheat bran decreased remarkably after the steam explosion (from 45.50% to 34.40%), while the percentage of soluble dietary fiber increased (from 3.09% to 7.47%), which is consistent with our previous study [17]. The high content of insoluble dietary fiber can cause a reduction in extrusion performance [23], while soluble dietary fiber provides a higher extrusion rate [24]. Therefore, the steam explosion pretreatment process, which degrades insoluble dietary fiber to soluble dietary fiber, dramatically improves wheat bran extrusion performance.

In addition, arabinoxylan is an essential component of wheat bran cell wall polysaccharides [25,26]. The water-extractable arabinoxylan content in water can be utilized as an indicator to investigate the degradation effect of steam explosion treatments on the cell wall [27,28]. It was clear that the water-extractable arabinoxylan content of the steam-exploded sample was much higher than that of the non-steam-exploded sample (by about 1567.4%). On one hand, the high water-extractable arabinoxylan content of steam-exploded wheat bran confirms the destructive effect of steam explosion on the wheat bran cell wall; on the other hand, this may facilitate its extrusion performance [29].

### 3.2. Extrusion Performance and Grindability of Samples

**Figure 3:**
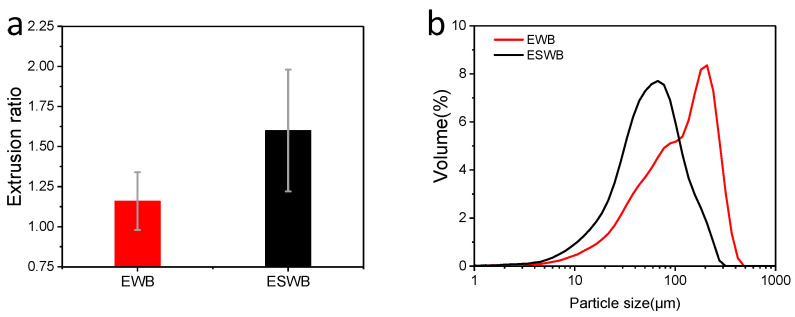
Macrostructural parameters (**a**) and particle size distributions (**b**) of extruded wheat bran (EWB) and extruded steam-exploded wheat bran (ESWB).

The extrusion ratio is the most important index used to measure the performance of extruded products. The extrusion performance of steam-exploded and non-steam-exploded samples is shown in Figure 3a. The steam-exploded wheat bran exhibited better extrusion performance, with an extrusion ratio of 1.60, while the non-steam-exploded wheat bran was almost unexpanded (extrusion ratio of 1.16). Moreover, steam explosion pretreatment reduced problems such as the clogging, smoking, and spraying of wheat bran during the extrusion process, which made the extrusion of wheat bran accessible. The above results indicate that the untreated wheat bran has almost no extrusion capacity, while steam explosion gives better extrusion properties to wheat bran.

To further characterize the expansibility of steam-exploded wheat bran, the grindability of extruded wheat bran samples was investigated. The particle size distribution of the two samples is shown in Figure 3b and Table 2. The particle size distribution (D90) of the two samples was quite different, and the steam explosion pretreatment resulted in a lower particle size distribution of the extruded wheat bran flour under the same grinding conditions. The causes of this can be traced to the extruded wheat bran, which contains a large number of internal voids that lead to its loose structure, with reduced mechanical strength. The mechanical strength of samples with high extrusion ratios can be reduced more significantly and thus are more susceptible to being ground into fine flour [30]. Thus, the enhancement of wheat bran extrusion properties by steam explosion pretreatment was demonstrated based on the fact that steam-exploded wheat bran was more likely to be ground into fine flour. In addition, the cell wall breakage ratios of extruded samples without and after pretreatment were 25.05% and 47.00%, respectively. This indicates that the steam explosion pretreatment facilitates the cell wall breakage rate in wheat bran flour, which could improve the release level of intracellular substances and is more conducive to improving the nutritional value of the food and promoting the absorption of nutrients by the human body.

### 3.3. Functional Properties of Samples

**Figure 4:**
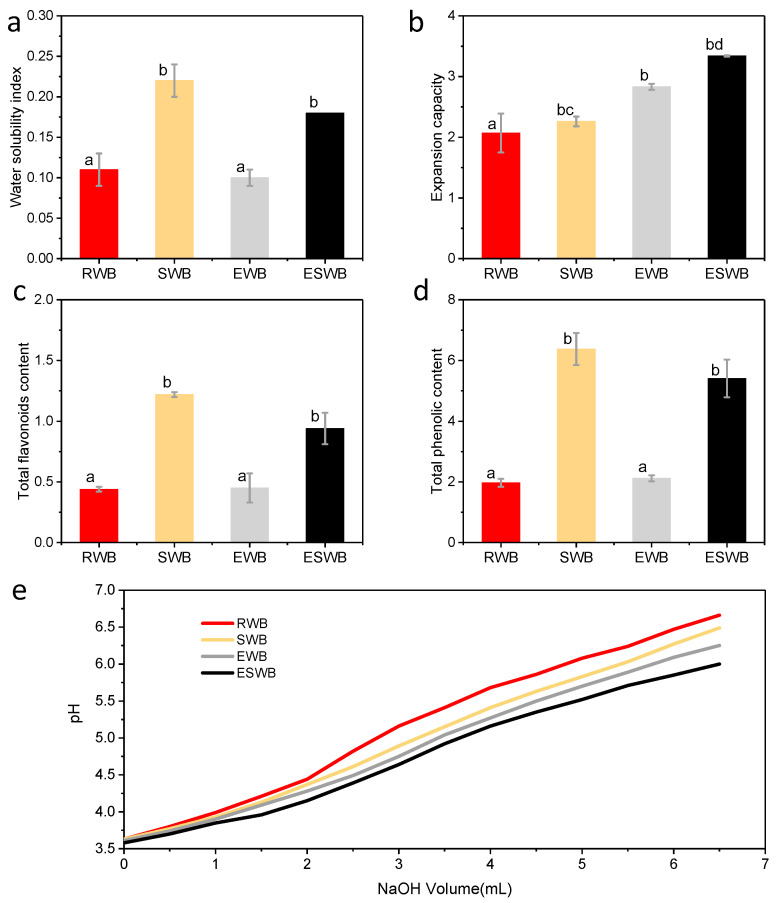
Water solubility index (**a**), expansion capacity (**b**), total flavonoid (**c**) and phenolic (**d**) contents, as well as cation exchange capacity (**e**) of raw wheat bran (RWB), steam-exploded wheat bran (SWB), extruded wheat bran (EWB), and extruded steam-exploded wheat bran (ESWB); means that are significantly different (*p* < 0.05) are indicated by different letters.

The water solubility index indicates the decomposition degree of sugar and fiber substances in the sample [31]. Higher solubility indicates that the sample contains fewer macromolecules and more substances that the human body can absorb more easily. In Figure 4a, the water solubility indices of wheat bran with different treatments are shown. The water solubility index of the samples treated with steam explosion (0.22 and 0.18 g/g) was significantly higher than those of the untreated samples (0.11 and 0.10 g/g), which could be attributed to the soluble pentosan content increase due to the disruption of the wheat bran cell wall through the steam explosion [17] (Figure 4a). The extrusion process loosens the structure of wheat bran and makes it easy to expand. Therefore, the expansion properties of the extrusion-treated wheat bran samples were all stronger than those of the untreated samples (Figure 4b).

Compared with the non-steam-exploded sample, the total flavonoid content and total phenolic content of steam-exploded wheat bran were increased by 177.27% and 223.85%, respectively (Figure 4c,d). This phenomenon may be due to the steam explosion disrupting the dense structure of the cell wall of wheat bran and promoting the dissolution of the flavonoid and phenolic compounds [17]. In addition, cell wall friction damage during the steam explosion process resulted in the release of conjugated phenolic compounds that were bound to polysaccharides or proteins [32]. It indicates that steam explosion treatment facilitates nutrient extraction from wheat bran and allows for the efficient utilization of wheat bran [33]. The total phenolic and flavonoid contents of extruded wheat bran were not significantly changed (Figure 4c,d). This may be due to the intracellular substance dissolution promoted in wheat bran cells by the extrusion process; the high temperature in this process resulted in the inactivation of phenolics and flavonoids, which counteracted each other [34].

Due to its side chain groups, such as carboxyl, hydroxyl, and amino groups, dietary fiber can reversibly exchange cations, lowering blood pressure [35,36]. As the content of NaOH in the system increased, its pH was raised (Figure 4b). From Equation (1), it can be seen that the larger volume of sodium hydroxide consumed the higher cation exchange capacity of the sample as the target pH was reached. After extrusion treatment, the steam-exploded wheat bran exhibited the highest sodium hydroxide demand, indicating its high cation exchange capacity. The enhanced cation exchange capacity of wheat bran may be due to a tremendous number of functional groups being exposed after the dual treatment of steam explosion and extrusion [37]. The above results indicate that steam explosion improves the puffing performance of wheat bran and enhances the functionality of the puffed product, thus promoting the functionalization of wheat bran.

## 4. Conclusions

In this study, steam explosion pretreatment was applied to improve the extrusion ratio of wheat bran. The steam explosion can promote the conversion of insoluble dietary fiber to soluble dietary fiber, increase the content of water-extractable arabinoxylan, as well as break the dense structure of the cell wall of wheat bran to facilitate the dissolution of flavonoid and phenolic compounds. Steam-exploded wheat bran exhibited a higher extrusion capacity than untreated samples. Furthermore, the grindability of the samples was further evidenced; the extrusion ratio of steam-exploded wheat bran was superior to that of raw wheat bran. Physicochemical properties such as the water solubility index, expansion capacity, and cation exchange capacity of the steam-exploded wheat bran after extrusion were enhanced compared to the other samples. In summary, this work demonstrates that steam explosion pretreatment upgrades the extrusion capacity of wheat bran, broadening the approach to the processing of wheat bran and boosting the potential of wheat bran applications in foods.

## Figures and Tables

**Table 1:** FTIR semi-quantitative analysis of RWB and SWB.

Band (cm^−1^)	Assignment	Relative Intensity	
		RWB	SWB
1041	COC stretching typical of glucan and xylan	2.3993	1.3404
1242	CO stretching vibration of acetyl group in the lignin	1.2049	1.0844
1519	Aromatic skeletal stretching	1.0000	1.0000
1650	CO stretching of amide I	1.6237	1.1483
2923	CH in cellulose and hemicellulose	1.2385	1.0782
3348	OH stretching and hydrogen bonds	1.8799	1.1769

**Table 2:** Particle size distribution of EWB and ESWB flour.

Samples	D10 (μm)	D50 (μm)	D90 (μm)	Cell Wall Breakage Ratio (%)
EWB	26.97 ± 0.60	109.15 ± 3.00	239.07 ± 4.33	25.05 ± 0.62
ESWB	17.07 ± 1.27	52.48 ± 2.40	126.79 ± 4.48	47.00 ± 1.71

## Data Availability

The data presented in this study.
